# VGLUT3 does not synergize GABA/glycine release during functional refinement of an inhibitory auditory circuit

**DOI:** 10.3389/fncir.2014.00140

**Published:** 2014-11-26

**Authors:** Daniel T. Case, Javier Alamilla, Deda C. Gillespie

**Affiliations:** ^1^Neuroscience Graduate Program, McMaster UniversityHamilton, ON, Canada; ^2^Department of Psychology, Neuroscience and Behaviour, McMaster UniversityHamilton, ON, Canada

**Keywords:** co-transmission, lateral superior olive, medial nucleus of trapezoid body, vesicular transport proteins, inhibitory synapses, development

## Abstract

The vesicular glutamate transporter 3 (VGLUT3) is expressed at several locations not normally associated with glutamate release. Although the function of this protein has been generally elusive, when expressed in non-glutamatergic synaptic terminals, VGLUT3 can not only allow glutamate co-transmission but also synergize the action of non-glutamate vesicular transporters. Interestingly, in the immature glycinergic projection between the medial nucleus of the trapezoid body (MNTB) and the lateral superior olive (LSO) of auditory brainstem, the transient early expression of VGLUT3 is required for normal developmental refinement. It has however been unknown whether the primary function of VGLUT3 in development of these inhibitory synapses is to enable glutamate release or to promote loading of inhibitory neurotransmitter through vesicular synergy. Using tissue from young mice in which *Vglut3* had been genetically deleted, we evaluated inhibitory neurotransmission in the MNTB-LSO pathway. Our results show, in contrast to what has been seen at adult synapses, that VGLUT3 expression has little or no effect on vesicular synergy at the immature glycinergic synapse of brainstem. This finding supports the model that the primary function of increased VGLUT3 expression in the immature auditory brainstem is to enable glutamate release in a developing inhibitory circuit.

## Introduction

The glycinergic projection from the medial nucleus of the trapezoid body (MNTB) to lateral superior olive (LSO) is a critical component of the neural circuit for localizing higher frequency sound sources (for review, see Tollin, 2003, though see also Jalabi et al., 2013). Although the MNTB-LSO pathway, with its precise, tonotopically organized circuitry and well-defined inputs, has been a model system for understanding inhibitory circuit refinement, the mechanisms of this synaptic refinement have yet to be elucidated, in part because immature MNTB-LSO synapses exhibit a complex neurotransmitter phenotype. First, as is common at other immature glycinergic synapses, MNTB terminals in the LSO use GABA transmission (Kotak et al., 1998; Nabekura et al., 2004), for unknown reasons (Gillespie and Kandler, 2009). Second, due to the transient expression of vesicular glutamate transporter 3 (VGLUT3), MNTB terminals also release glutamate during the first postnatal week (Gillespie et al., 2005), a period characterized by major synaptic refinement (Kim and Kandler, 2003). As hearing onset does not occur until about postnatal day 10 (P10), this circuit refinement is understood to be directed by neural activity spontaneously generated in the cochlea (Tritsch et al., 2007), and the expression of VGLUT3 is required for normal refinement (Noh et al., 2010). The precise role of VGLUT3 in this refinement has not been elucidated.

The function of VGLUT3 in the nervous system generally has been elusive. Although originally identified by sequence homology with its more common family members *Vglut1 and Vglut2, Vglut3* has a distinctly different expression pattern and likely function. First, VGLUT3 exhibits a biphasic temporal expression pattern, with intense, transient expression in immature cerebellum and brainstem. Additionally, in both neonatal and adult tissue, VGLUT3 is notably expressed at “non-glutamatergic” release sites (Fremeau et al., 2002; Gras et al., 2002, 2005; Herzog et al., 2004). This seemingly ectopic expression of VGLUT3 naturally led to an initial hypothesis that a primary function might be to enable glutamate release from non-canonical glutamate synapses and/or from astrocytes. More recently, however, another role has come to light. In particular, in the striatum and raphe nucleus of adult, VGLUT3 is targeted to cholinergic and serotonergic vesicles where it synergizes the action of vesicular transporters for acetylcholine and serotonin, increasing both rate and degree of vesicle filling (Gras et al., 2008; Amilhon et al., 2010; for review, see El Mestikawy et al., 2011).

In the MNTB-LSO pathway, the working model for the role of VGLUT3 in functional refinement has been that depolarizing GABA and/or glycine at the immature MNTB-LSO synapse relieves Mg^++^-block at NMDA receptors that, upon their activation by glutamate co-transmission, mediate activity-dependent plasticity (Kandler and Friauf, 1995; Kotak et al., 1998; Ehrlich et al., 1999; Kalmbach et al., 2010; Case and Gillespie, 2011). A central assumption of this model is that VGLUT3 is expressed in the developing auditory brainstem primarily to enable glutamate release from immature glycinergic synapses. The possibility that VGLUT3 could synergize packaging of GABA/glycine vesicles in immature MNTB terminals, however, gives rise to an alternate hypothesis for why developmental refinement here requires VGLUT3. In the alternate model, at what would otherwise be a sluggish immature synapse, VGLUT3 boosts GABA/glycinergic transmission, activating L-type voltage-gated Ca^++^ channels (Kullmann et al., 2002) that could lead to downstream plasticity.

If VGLUT3 permits or directs refinement of MNTB-LSO synapses by boosting GABA/glycinergic vesicle loading, we would expect loss of VGLUT3 to cause abnormal GABA/glycinergic transmission during the period of major circuit refinement. Using whole-cell recordings in tissue from genetically altered mice, we found that VGLUT3 expression does not synergize GABA/glycine vesicle loading in the developing MNTB-LSO circuit.

## Materials and methods

All procedures adhered to Canadian Council on Animal Care guidelines and were previously approved by the Animal Research Ethics Board of McMaster University. Mice heterozygous (+/−) for *Vglut3* (gift of S. El Mestikawy) were bred on site to yield litters containing wild-type (WT), heterozygous (het), and knockout (KO) mice. Genotypes were determined after all other procedures had been performed, to ensure that slice preparation and electrophysiology were always performed blind to *Vglut3* expression.

Pups age postnatal day 4–5 (P4–5; day of birth is P0) were anesthetized and quickly decapitated, and the brains were removed into ice-cold artificial cerebrospinal fluid (ACSF, pH 7.2) containing (in mM): 125 NaCl, 1 MgSO_4_, 5 KCl, 1.25 KH_2_PO_4_, 10 dextrose, 26 NaHCO_3_, 2 CaCl_2_, 1 ascorbic acid, 1 kynurenic acid. Tail tissue for subsequent genotype determination was collected at sacrifice. Brainstem slices (300 µm) containing the MNTB and LSO were allowed to recover at room temperature for ≥1 h in a humidified, oxygenated, interface chamber. Slices were transferred to a recording chamber where they were continuously perfused with ACSF at elevated temperature (32–35°C) and superfused with 95% O_2_/5% CO_2_. Perfusion ACSF was identical to slicing ACSF, with the addition of 0.3 mM ascorbic acid and 0.5 mM D-glutamine.

Recording electrodes (1–4 MΩ) were filled with a Cs-gluconate solution containing (in mM): 64 D-gluconic acid, 64 CsOH, 11 EGTA, 56 CsCl, 1 MgCl_2_, 1 CaCl_2_, 10 HEPES, 0.3 GTP-Na, 4 ATP-Mg, 0.1 mM spermine (Acros Organics). In several cases, the internal solution also contained 0.5% biocytin for histological verification of cell type; in some instances QX-314 (5 mM; Tocris) was added to the internal solution to increase patch stability. Principal cells in the medial and middle limbs of the LSO were visually identified under IR-DIC by their morphology and orientation and were patched in whole-cell mode. Recordings were sampled at 10 kHz, and filtered at 5 kHz. Series resistance was compensated by 80% with < 10 µs lag, and recordings were discarded if series resistance changed by ≥15%. All recordings were made at a holding potential of −60 mV. Data were saved for offline analysis using MiniAnalysis (Synaptosoft), Clampfit (Molecular Devices) or custom Matlab programs, and are presented as mean +/− S.E.M.

In each slice, a stimulating electrode was placed at the lateral edge of the MNTB, the minimum intensity that reliably elicited a response was determined, and stimulus intensity thereafter was unaltered throughout the experiment. Within pulse trains, the peak current amplitude for each pulse was measured relative to current immediately before the pulse; all paired-pulse ratios (PPRs) are reported with the amplitude of the first response in the denominator.

To estimate quantal content and probability of release, we delivered 20 electrical stimuli at 100 Hz and normalized the amplitude of each response in the train to the amplitude of the first response. We then performed a linear regression on the last 6 points in the cumulative response amplitude curve and extrapolated to time = 0 (response 1) to estimate the size of the readily releasable pool of vesicles multiplied by quantal amplitude (N_q_) (Inchauspe et al., 2007), and thence to estimate probability of release.

Spontaneous miniature GABA/glycinergic events are uncommon in the immature LSO. To increase the probability of observing miniature GABA/glycinergic events, we delivered high-frequency stimulation (20 pulses at 100 Hz) to MNTB fibers, or replaced CaCl_2_ in the ACSF with SrCl_2_ (2 mM) to promote asynchronous vesicular release. Miniature events were identified, individually verified, and analyzed in MiniAnalysis. For comparison and verification, a subset of miniature events was also analyzed in Clampfit. As results from the two analyses showed no differences, all results shown here were analyzed in MiniAnalysis.

DNA was isolated from tail tissue obtained at sacrifice, and polymerase chain reaction (PCR) reactions using *VGlut3* WT (278 bp) and *VGlut3* KO cassette (604 bp) primers (Gras et al., 2008; Mobix Lab, McMaster University) were completed in separate tubes in a Peltier Thermal Cycler (Dyad DNA Engine). Following the PCR reaction, primer bands were identified using gel electrophoresis.

## Results

If the vesicular synergy model holds true in the immature MNTB-LSO pathway, we would expect differing levels of *Vglut3* to correlate with differences in GABA/glycinergic transmission, in particular for measures of short-term plasticity, recovery from short-term depression, and quantal size. For example, if VGLUT3 promotes faster vesicle reloading, VGLUT3-expressing synapses might be expected to recover more quickly from short-term depression. More specifically, we predicted that quantal size and hence the amplitude of miniature synaptic events would be larger at MNTB-LSO synapses expressing VGLUT3.

Normal developmental refinement in the murine MNTB-LSO pathway, which is characterized by changes in quantal size, quantal content, and probability of release (Kim and Kandler, 2010), is perturbed in *VGlut3^−/−^* mice (Noh et al., 2010). Therefore, to minimize the likelihood of measuring differences that had resulted from an ultimate effect of VGLUT3 on synaptic refinement rather than a proximal effect on vesicular synergy, we restricted our recordings to tissue from animals P4-P5. At this age in LSO, VGLUT3 levels and glutamate release are relatively high and the synaptic changes associated with the major period of developmental refinement have just begun. To avoid possible bias, all tissue collection, recordings, and analyses were performed blind to genotype.

### Vglut3 expression does not affect GABA/glycine release upon 50 Hz stimulation

**Figure 1:**
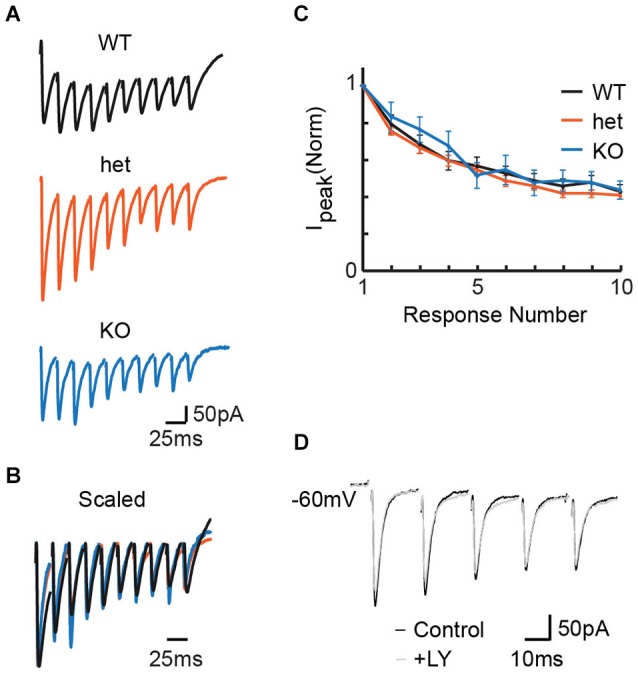
**Stimulation at 50 Hz in the MNTB revealed no effect of genotype on release properties in the LSO**. **(A)** Representative responses to 50 Hz stimulation for WT (P4), het (P5), and KO (P4) tissue. **(B)** Example traces from **(A)**, scaled for comparison. Traces were first scaled to force all genotypes to have the same value for the amplitude of the first response; then, within each train, the baseline for each response was reset to zero (i.e., baseline for first-response). **(C)** No differences appeared between WT (*n* = 20), het (*n* = 49), and KO (*n* = 9) mice for paired-pulse ratios (**C**; *p* = 0.32, see text). **(D)** Application of the mGluR antagonist LY 341 495 had no effect on PPR in slices expressing VGLUT3. Example P5 WT slice.

We first wanted to determine whether PPRs differ between *VGlut3* WT, heterozygous (het), and KO mice. We made whole-cell recordings in the LSO while delivering pulse trains to MNTB fibers at frequencies previously shown to cause short-term depression at immature MNTB-LSO synapses (Kim and Kandler, 2010; Case and Gillespie, 2011). Most cells exhibited depression consistent with previous results; the small number of anomalous cells that exhibited facilitation (*n* = 1 WT, 4 het, 2 KO) were excluded from analysis. Representative responses (for PPR) to stimulation at 50 Hz in the MNTB (Figures 1A,B) show no influence of genotype on PPR. We note that the responses depicted here were chosen by selecting for each condition the response with median PPR. It happens that the WT recording with median PPR response (shown) exhibited a background tonic current not seen in the median responses for the het and KO conditions. However, this tonic current is atypical; it was not characteristic, it did not occur more often in any particular genotype, and it did not affect PPR (data not shown). For the population of cells stimulated at 50 Hz, we found no effect of genotype on PPR (Figure 1C; PPRs: WT = 0.79 +/− 0.05, *n* = 21; het = 0.75 +/− 0.02, *n* = 49; KO = 0.83 +/− 0.08, *n* = 9; Kruskal-Wallis test: *p* = 0.32). Co-expression of VGLUT3 with other vesicular transporters has been shown to increase the rate and extent to which synaptic vesicles are filled (Gras et al., 2008; Amilhon et al., 2010). If VGLUT3 synergizes vesicle filling and if vesicle filling is a rate-limiting step, we would expect synapses from KO tissue to follow high frequency stimulation less well than those from WT or het tissue. Thus, these results do not support vesicular synergy between VGLUT3 and the GABA/glycine vesicular transporter (VIAAT/VGAT).

In the neonatal rat LSO, repetitive stimulation of the glutamatergic afferents from the ipsilateral cochlear nucleus can cause mGluR-mediated modulation of inhibitory neurotransmission at MNTB terminals (Nishimaki et al., 2007). To ask whether the presumably smaller amount of glutamate released by MNTB terminals could similarly modulate release properties, we compared pulse trains before and after the application of the mGluR2/3 antagonist LY 341 495 (Tocris) in a separate set of 3 WT slices (example shown in Figure 1D). Because blocking mGluRs did not affect paired pulse ratios or current amplitudes in any instance, we did not include the antagonist in further studies.

### Vglut3 expression does not affect GABA/glycine release upon 100 Hz stimulation

**Figure 2:**
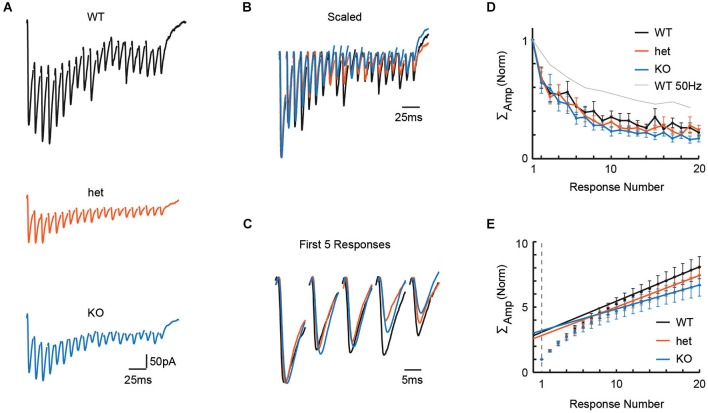
**Stimulation at 100 Hz in the MNTB revealed no effect of genotype on release properties in the LSO**. To more stringently test for differences in release properties, we delivered 20 pulses at 100 Hz in tissue from WT (*n* = 8), het (*n* = 9), and KO (*n* = 8) mice. **(A)** Representative responses to 100 Hz trains of 20 pulses for WT (P4), het (P4), and KO (P4) tissue. **(B)** Example traces from **(A)**, scaled for comparison. Traces were first scaled to force all genotypes to have the same value for first-response-amplitude; then, within each train, the baseline for each response was reset to zero (i.e., baseline for first-response). **(C)** First 5 responses from examples in **(B)**, enlarged for clarity. **(D,E)** No differences were apparent between WT (*n* = 8), het (*n* = 9) and KO (*n* = 8) tissue for paired-pulse ratios (**D**; *p* = 0.75, see text) or cumulative event amplitudes (**E**; *p* = 0.28, see text). Probability of release, as estimated using these cumulative amplitude functions, was also similar among genotypes (see text).

Adult neurons in the auditory brainstem achieve high rates of neural activity, and so, even in young tissue, it is possible that 50 Hz stimulation is simply too slow to test the system. In a new set of slices, we therefore stressed the synapse further by delivering stimuli at 100 Hz to the MNTB while recording in the LSO. Representative recordings from WT, het, and KO mice following 100 Hz stimulation are shown in Figure 2A, and these traces are shown overlaid in Figures 2B,C (normalized to the highest amplitude recording, the WT cell, in Figure 2A). With the faster 100 Hz stimulation, we still found no effect of genotype on paired-pulse depression (Figure 2D; PPRs: WT = 0.68 +/− 0.09, *n* = 8; het = 0.68 +/− 0.08, *n* = 9; KO = 0.64 +/− 0.09, *n* = 8; Kruskal-Wallis: *p* = 0.75), or on cumulative normalized amplitude of the pulse train (Figure 2E; cumulative amplitudes of 20 responses: WT = 8.06 +/− 0.86, *n* = 8; het = 7.43 +/− 0.74, *n* = 9; KO = 6.68 +/− 0.81, *n* = 8; Kruskal-Wallis: *p* = 0.28).

From the same cells, using cumulative amplitude to estimate quantal content N_q_ and probability of release (see Methods), we found similar estimates for N_q_ and release probability between genotypes (Figure 2E; N_q_: WT = 3.1 +/− 0.3; het = 2.8 +/− 0.3; KO = 3.2 +/− 0.4; Kruskal-Wallis test, *p* = 0.74; release probability: WT = 0.35 +/− 0.04; het = 0.39 +/− 0.05; KO = 0.35 +/− 0.05; Kruskal-Wallis test, *p* = 0.71). Together, these results argue that *VGlut3* expression does not affect release properties for GABA/glycine at MNTB terminals.

### Vglut3 expression does not affect recovery from depression following 50 Hz stimulation

**Figure 3:**
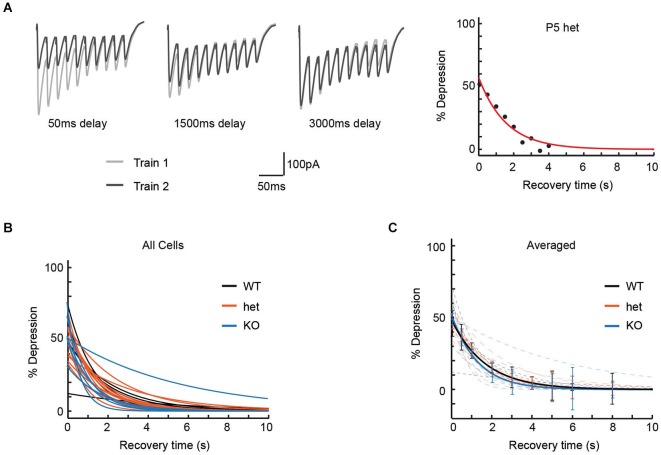
**Recovery from paired-pulse depression was unaffected by genotype**. To determine the time required for MNTB-LSO synapses to recover from paired-pulse depression, we delivered pairs of 50 Hz trains of 10 pulses each, with each pair of trains separated by intervals ranging from 50 ms to 12 s. **(A)** Left: Three representative responses used to determine recovery time for a P5 het neuron. In each panel, Train 1 comprises the responses to the initial pulse train, and Train 2 the responses to the second pulse train of the pair, delivered after the interval shown. Right: Percent depression as a function of recovery interval (8 intervals, 50 ms to 4 s) for the cell shown at the left. Recovery time constant = 1.56 s; R-square of curve fit = 0.94. **(B)** Exponential fits for recovery from depression are shown for each cell thus examined (*n* values: WT = 5, het = 12, KO = 6). **(C)** Average of recovery curves (bold) for each genotype, with individual recovery curves from **(B)** in background (dashed, desaturated curves). Genotype had no effect on time to recovery from paired-pulse depression (Recovery time constant: WT = 2.4 +/− 0.7 s, het = 1.7 +/− 0.2 s, KO = 1.9 +/− 0.8 s; Kruskal-Wallis test, *p* = 0.32).

Though our pulse train experiments had failed to show obvious effects of *VGlut3* expression on release properties, we considered the possibility that differences in vesicle re-filling might affect synaptic transmission on a longer timescale, and that this might be observed as an effect on recovery from depression. To test this model, we delivered two 50 Hz 10-pulse trains, separating by varying intervals (Dittman and Regehr, 1998) in order to estimate the time required for neurotransmission to recover to its original level (Figure 3A).

Cells were included in this analysis if we were able to test at least 8 recovery intervals, including one of at least 4 s. In practice, using these criteria ensured that 90% or greater recovery was attained for all cells analyzed. Percent depression as a function of recovery interval was then fit to an exponential (individual cells, Figure 3B; averages, Figure 3C). Neither average time to 90% recovery (WT: 3.2 +/− 0.7 s, *n* = 5; het: 2.9 +/− 0.2 s, *n* = 12; KO: 3.0 +/− 1.0 s, *n* = 6; Kruskal-Wallis test, *p* = 0.76) nor exponential time constants for recovery (recovery time constants: WT = 2.4 +/− 0.7 s, het = 1.7 +/− 0.2 s, KO = 1.9 +/− 0.8 s; Kruskal-Wallis test, *p* = 0.32) differed among genotypes.

### Vglut3 expression does not affect size of GABA/glycine miniature events

**Figure 4:**
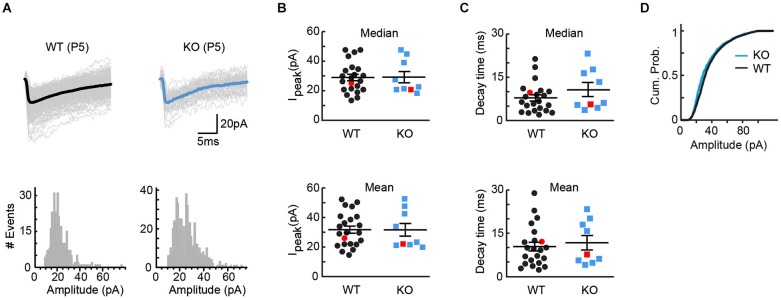
**Quantal size, as assessed by miniature events, was unaffected by genotype**. **(A)** Example miniature events for cells from WT (left, P5, 537 events, mean = 25.9 +/− 0.4 pA, median = 25.0 pA) and KO (right, P5, 326 events, mean = 22.1 +/− 0.5 pA, median = 20.8 pA) tissue. Bold trace is the average miniature event for each cell. Event histograms for each cell are included below the example traces. **(B)** No differences were observed between WT (*n* = 22) and KO (*n* = 9) tissue for either mean or median peak amplitude (means: WT = 31.8 +/− 2.4 pA, KO = 31.6 +/− 4.3 pA; *t*-test *p* = 0.98; medians: WT = 29.0 +/− 2.2 pA, KO = 29.2 +/− 3.8 pA; *t*-test *p* = 0.95). Red markers indicate example cells in **(A)**. **(C)** For the cells shown in **(B)**, no differences were observed between WT and KO tissue for either mean or median decay times (means: WT = 10.3 +/− 1.5 ms, KO = 11.7 +/− 2.5 ms; *t*-test, *p* = 0.65; medians: WT = 7.8 +/− 1.1 ms, KO = 10.6 +/− 2.4 ms; *t*-test, *p* = 0.23). Red markers indicate example cells in **(A)**. **(D)** Cumulative probabilities for all miniature amplitudes, pooled within genotype, for the cells shown in **(B,C)**.

VGLUT3 has been shown to increase not only the rate, but also the amount of transmitter loading into synaptic vesicles (Gras et al., 2008). If VGLUT3 increases the concentration of GABA/glycine in vesicles at MTNB terminals, we would expect KO tissue to exhibit smaller responses to quantal release. To test this model, we compared the amplitudes of miniature GABA/glycinergic events recorded from WT and KO tissue. Because spontaneous vesicular release is uncommon in the immature LSO, we artificially increased release of single vesicles by either (1) first delivering brief high frequency stimulation to the MNTB-LSO pathway, or (2) adding Sr^++^ to the perfusate to desynchronize vesicular release in response to single stimuli (Bekkers and Clements, 1999). Miniature events were collected in the 20 s following the stimulus, and the individual events were analyzed. Cells were always allowed to recover at least 20 s before a new stimulus. In the examples of Figure 4A, no effect of genotype on mean event amplitude is seen (WT: 537 events, mean amplitude = 25.9 +/− 0.4 pA, median amplitude = 25.0 pA; KO: 326 events, mean amplitude = 22.1 +/− 0.5 pA, median amplitude = 20.8 pA). This similarity in event amplitude held true across all cells (Figure 4B; WT: average mean amplitude = 31.8 +/− 2.4 pA, average median amplitude = 29.0 pA+/− 2.2 pA, *n* = 22; KO: average mean amplitude = 31.6 +/− 4.3 pA, average median amplitude = 29.2 pA+/− 3.8 pA, *n* = 9; *t*-test for means, *p* = 0.98; *t*-test for medians, *p* = 0.95). Comparison of current decay times also yielded no significant difference between genotypes (Figure 4C; WT: average mean decay = 10.3 +/− 1.5 ms, average median decay = 7.8 +/− 1.1 ms; KO: average mean decay = 11.7 +/− 2.5 ms, average median decay = 10.6 +/− 2.4 ms; *t*-test for means, *p* = 0.65; *t*-test for medians, *p* = 0.23), and even the cumulative probabilities found by pooling all miniatures within genotype showed only the suggestion of a difference (Figure 4D). Together, these results provide further evidence that *VGlut3* expression does not appreciably influence GABA/glycine vesicle loading at immature MNTB terminals.

## Discussion

*VGlut3* is expressed in select populations of typically non-glutamatergic neurons. At adult synaptic terminals, VGLUT3 can increase both rate and level of neurotransmitter packing in cholinergic, serotonergic, and GABAergic vesicles in the striatum, raphe nuclei and hippocampus (Gras et al., 2008; Amilhon et al., 2010; Zander et al., 2010), and at least in the striatum VGLUT3 is required for normal cholinergic transmission (Nelson et al., 2014). To ask whether the transient expression of *VGlut3* during early postnatal development plays a similar role in synergizing GABA/glycine transmission at immature MNTB terminals in the LSO, we made whole-cell patch-clamp recordings from *VGlut3* WT, heterozygous and KO mice. The similarity among tissue from WT, het, and KO mice for PPRs and time to recovery from synaptic depression argues against a primary effect of VGLUT3 on rate of vesicle filling. The similarity in amplitude of miniature GABA/glycinergic events across genotype further argues against a model in which VGLUT3 increases level of vesicle filling. As the only other known function for VGLUT3 is to enable glutamate release, our results lend further support to the model that normal developmental refinement in the MNTB-LSO pathway requires VGLUT3 because refinement requires glutamate release. This model still needs to be tested explicitly.

We note that VGLUT3 may have exerted a nuanced effect on GABA/glycine transmission in our samples (e.g., Figure 4D), but if so, it was a small effect, and one that might well have occurred if already by P4–5 a subset of synapses had undergone more maturation in WT than in KO mice (Kim and Kandler, 2010; Noh et al., 2010). It has been nicely demonstrated that the window for synaptic refinement in the rat MNTB-LSO pathway is between P3 and P8 (Kim and Kandler, 2003), and in mice, we expect this period to be the same or perhaps shifted a day earlier. While in theory our question could be addressed by analyzing tissue collected before the synaptic refinement begins, VGLUT3 expression increases enough between P1 and P5 to cause problems for such an analysis. We note also that if VGLUT3 normally induces vesicular synergy that is followed by a compensatory reduction in quantal content (Daniels et al., 2004) we might miss an effect of VGLUT3 on miniature synaptic events. Nevertheless, whatever advantage VGLUT3 may provide to GABA/glycine neurotransmission, clearly that effect is small relative to the transformative effect of enabling glutamate release from immature inhibitory terminals. In sum, these results suggest that VGLUT3 plays qualitatively different roles at neonatal and adult inhibitory synapses, and in the immature auditory brainstem the likely primary function of VGLUT3 is to enable glutamate release.

Although *VGlut3* is already highly expressed in the superior olive at P4–5, its expression continues to increase until about P9–10, after which it declines rapidly (Gras et al., 2005). This raises the question of whether VGLUT3-induced vesicular synergy could be more prominent at P9–P10 and we did not observe it simply because we tested at P4–5. In fact, it would be difficult to disentangle demonstrated differences in miniature event amplitudes between WT and *VGlut3* KO mice at P9–12 (Noh et al., 2010), which are understood to reflect normal developmental strengthening due to postsynaptic effects (Kim and Kandler, 2010), from a synergistic effect. Nevertheless, even if any part of these differences at P9–12 could be attributed to a larger role for VGLUT3 in vesicular synergy, P9 is already past the window for functional refinement in the MNTB-LSO pathway (Kim and Kandler, 2003); hence, vesicular synergy found at P9 could not be a significant contributor to refinement.

Another possibility that could have obscured a vesicular synergy effect in our study is if loss of VGLUT3 results in compensatory presynaptic expression of VGLUT1 or VGLUT2 or increased postsynaptic expression of GABA_A_ and/or glycine receptors. This seems unlikely. In vesicular synergy studies with *VGlut3* KO mice, compensatory increases in glutamate transporters have not been seen (Gras et al., 2008). Furthermore, if another vesicular glutamate transporter were upregulated, glutamate release should still be seen in the MNTB-LSO pathway of *VGlut3* KO mice, whereas in fact it disappears (Noh et al., 2010). Finally, while it is possible that compensatory changes in the expression of other, unidentified, synaptic proteins could have prevented our identification of a vesicular synergy effect, once again, that hypothetical compensatory effect is insufficient to rescue refinement (Noh et al., 2010), which further supports the conclusion that supporting vesicular synergy is not the critical role VGLUT3 plays in MNTB-LSO pathway refinement.

Normally considered a rare protein, VGLUT3 exhibits a striking expression profile in the developing superior olive, increasing dramatically from birth until about P10 and then declining to nearly undetectable levels (Blaesse et al., 2005; Cooper and Gillespie, 2011). As this transient expression most probably reflects a distinctly developmental role for VGLUT3 at immature inhibitory synapses in the superior olive, our results do not contradict but rather complement those reported at adult synapses. Indeed, whereas mature expression of VGLUT3 can suppress excitability (Zander et al., 2010) and support synergistic packaging of other neurotransmitters (Gras et al., 2008; Amilhon et al., 2010), the early expression of VGLUT3 in the auditory brainstem is required for normal developmental circuit refinement (Noh et al., 2010).

The refinement for which VGLUT3 is required in the rodent LSO occurs during the first postnatal week (Kim and Kandler, 2003, 2010), a period during which the brainstem receives no acoustically driven inputs. Activity-dependent refinement must therefore rely upon the activity generated spontaneously in the developing cochlea (Tritsch et al., 2007, 2010), and indeed it has recently been shown that altering the temporal statistics of this activity affects circuit refinement (Clause et al., 2014). As the mechanisms underlying refinement in this inhibitory circuit are still unknown, the role of VGLUT3 has inspired speculation. In one proposed role, VGLUT3 enables glutamate co-transmission to activate NMDA receptors. In the alternate proposed role, VGLUT3 boosts GABA/glycinergic transmission, which in turn depolarizes NMDA receptors or activates voltage-gated Ca^++^ channels. Our results argue against a primary function of VGLUT3 in promoting loading of GABA/glycine into synaptic vesicles in the developing brainstem and highlight the importance of establishing a robust model for mechanistic studies of synaptic plasticity and the role of VGLUT3 at this glycinergic synapse.

## Conflict of interest statement

The authors declare that the research was conducted in the absence of any commercial or financial relationships that could be construed as a potential conflict of interest.

## References

[B1] Amilhon B. Lepicard E. Renoir T. Mongeau R. Popa D. Poirel O.. (2010). VGLUT3 (vesicular glutamate transporter type 3) contribution to the regulationof serotonergic transmission and anxiety. J. Neurosci. 30, 2198–2210. 10.1523/jneurosci.5196-09.2010 20147547 PMC6634029

[B2] Bekkers J. M. Clements J. D. (1999). Quantal amplitude and quantal variance of strontium-induced asynchronous EPSCs in rat dentate granule neurons. J. Physiol. 516, 227–248. 10.1111/j.1469-7793.1999.227aa.x 10066937 PMC2269216

[B3] Blaesse P. Ehrhardt S. Friauf E. Nothwang H. G. (2005). Developmental pattern of three vesicular glutamate transporters in the rat superior olivary complex. Cell Tissue Res. 320, 33–50. 10.1007/s00441-004-1054-8 15714284

[B4] Case D. T. Gillespie D. C. (2011). Pre- and postsynaptic properties of glutamatergic transmission in the immature inhibitory MNTB-LSO pathway. J. Neurophysiol. 106, 2570–2579. 10.1152/jn.00644.2010 21832038

[B5] Clause A. Kim G. Sonntag M. Weisz C. J. Vetter D. E. Rbsamen R.. (2014). The precise temporal pattern of prehearing spontaneous activity is necessary for tonotopic map refinement. Neuron 82, 822–835. 10.1016/j.neuron.2014.04.001 24853941 PMC4052368

[B6] Cooper A. P. Gillespie D. C. (2011). Synaptotagmins I and II in the developing rat auditory brainstem: synaptotagmin I is transiently expressed in glutamate-releasing immature inhibitory terminals. J. Comp. Neurol. 519, 2417–2433. 10.1002/cne.22634 21456023

[B7] Daniels R. W. Collins C. A. Gelfand M. V. Dant J. Brooks E. S. Krantz D. E.. (2004). Increased expression of the Drosophila vesicular glutamate transporter leads to excess glutamate release and a compensatory decrease in quantal content. J. Neurosci. 24, 10466–10474. 10.1523/jneurosci.3001-04.2004 15548661 PMC6730318

[B8] Dittman J. S. Regehr W. G. (1998). Calcium dependence and recovery kinetics of presynaptic depression at the climbing fiber to Purkinje cell synapse. J. Neurosci. 18, 6147–6162. 9698309 10.1523/JNEUROSCI.18-16-06147.1998 PMC6793194

[B9] Ehrlich I. Lohrke S. Friauf E. (1999). Shift from depolarizing to hyperpolarizing glycine action in rat auditory neurones is due to age-dependent Cl- regulation. J. Physiol. 520, 121–137. 10.1111/j.1469-7793.1999.00121.x 10517806 PMC2269580

[B10] El Mestikawy S. Wallén-Mackenzie A. Fortin G. M. Descarries L. Trudeau L. E. (2011). From glutamate co-release to vesicular synergy: vesicular glutamate transporters. Nat. Rev. Neurosci. 12, 204–216. 10.1038/nrn2969 21415847

[B11] Fremeau R. T. Jr. Burman J. Qureshi T. Tran C. H. Proctor J. Johnson J.. (2002). The identification of vesicular glutamate transporter 3 suggests novel modes of signaling by glutamate. Proc. Natl. Acad. Sci. U S A 99, 14488–14493. 10.1073/pnas.222546799 12388773 PMC137910

[B12] Gillespie D. C. Kandler K. (2009). “GABA, glycine and glutamate co-release at developing inhibitory synapses,” in Co-Existence and Co-Release of Classical Neurotransmitters, ed Gutierrez R. (New York: Springer Verlag), 55–80.

[B13] Gillespie D. C. Kim G. Kandler K. (2005). Inhibitory synapses in the developing auditory system are glutamatergic. Nat. Neurosci. 8, 332–338. 10.1038/nn1397 15746915

[B14] Gras C. Amilhon B. Lepicard E. M. Poirel O. Vinatier J. Herbin M.. (2008). The vesicular glutamate transporter VGLUT3 synergizes striatal acetylcholine tone. Nat. Neurosci. 11, 292–300. 10.1038/nn2052 18278042

[B15] Gras C. Herzog E. Bellenchi G. C. Bernard V. Ravassard P. Pohl M.. (2002). A third vesicular glutamate transporter expressed by cholinergic and serotoninergic neurons. J. Neurosci. 22, 5442–5451. 12097496 10.1523/JNEUROSCI.22-13-05442.2002 PMC6758212

[B16] Gras C. Vinatier J. Amilhon B. Guerci A. Christov C. Ravassard P.. (2005). Developmentally regulated expression of VGLUT3 during early post-natal life. Neuropharmacology 49, 901–911. 10.1016/j.neuropharm.2005.07.023 16182324

[B17] Herzog E. Gilchrist J. Gras C. Muzerelle A. Ravassard P. Giros B.. (2004). Localization of VGLUT3, the vesicular glutamate transporter type 3, in the rat brain. Neuroscience 123, 983–1002. 10.1016/j.neuroscience.2003.10.039 14751290

[B18] Inchauspe C. G. Forsythe I. D. Uchitel O. D. (2007). Changes in synaptic transmission properties due to the expression of N-type calcium channels at the calyx of Held synapse of mice lacking P/Q-type calcium channels. J. Physiol. 584, 835–851. 10.1113/jphysiol.2007.139683 17823210 PMC2277003

[B19] Jalabi W. Kopp-Scheinpflug C. Allen P. D. Schiavon E. DiGiacomo R. R. Forsythe I. D.. (2013). Sound localization ability and glycinergic innervation of the superior olivary complex persist after genetic deletion of the medial nucleus of the trapezoid body. J. Neurosci. 33, 15044–15049. 10.1523/jneurosci.2604-13.2013 24048834 PMC3858601

[B20] Kalmbach A. Kullmann P. H. Kandler K. (2010). NMDAR-mediated calcium transients elicited by glutamate co-release at developing inhibitory synapses. Front. Synaptic Neurosci. 2:27. 10.3389/fnsyn.2010.00027 21423513 PMC3059663

[B21] Kandler K. Friauf E. (1995). Development of glycinergic and glutamatergic synaptic transmission in the auditory brainstem of perinatal rats. J. Neurosci. 15, 6890–6904. 7472446 10.1523/JNEUROSCI.15-10-06890.1995 PMC6578015

[B22] Kim G. Kandler K. (2003). Elimination and strengthening of glycinergic/GABAergic connections during tonotopic map formation. Nat. Neurosci. 6, 282–290. 10.1038/nn1015 12577063

[B23] Kim G. Kandler K. (2010). Synaptic changes underlying the strengthening of GABA/glycinergic connections in the developing lateral superior olive. Neuroscience 171, 924–933. 10.1016/j.neuroscience.2010.09.054 20888399 PMC2987552

[B24] Kotak V. C. Korada S. Schwartz I. R. Sanes D. H. (1998). A developmental shift from GABAergic to glycinergic transmission in the central auditory system. J. Neurosci. 18, 4646–4655. 9614239 10.1523/JNEUROSCI.18-12-04646.1998 PMC6792682

[B25] Kullmann P. H. Ene F. A. Kandler K. (2002). Glycinergic and GABAergic calcium responses in the developing lateral superior olive. Eur. J. Neurosci. 15, 1093–1104. 10.1046/j.1460-9568.2002.01946.x 11982621 PMC4120099

[B26] Nabekura J. Katsurabayashi S. Kakazu Y. Shibata S. Matsubara A. Jinno S.. (2004). Developmental switch from GABA to glycine release in single central synaptic terminals. Nat. Neurosci. 7, 17–23. 10.1038/nn1170 14699415

[B27] Nelson A. B. Bussert T. G. Kreitzer A. C. Seal R. P. (2014). Striatal cholinergic neurotransmission requires VGLUT3. J. Neurosci. 34, 8772–8777. 10.1523/jneurosci.0901-14.2014 24966377 PMC4069355

[B28] Nishimaki T. Jang I. S. Ishibashi H. Yamaguchi J. Nabekura J. (2007). Reduction of metabotropic glutamate receptor-mediated heterosynaptic inhibition of developing MNTB-LSO inhibitory synapses. Eur. J. Neurosci. 26, 323–330. 10.1111/j.1460-9568.2007.05656.x 17623021

[B29] Noh J. Seal R. P. Garver J. A. Edwards R. H. Kandler K. (2010). Glutamate co-release at GABA/glycinergic synapses is crucial for the refinement of an inhibitory map. Nat. Neurosci. 13, 232–238. 10.1038/nn.2478 20081852 PMC2832847

[B30] Tollin D. J. (2003). The lateral superior olive: a functional role in sound source localization. Neuroscientist 9, 127–143. 10.1177/1073858403252228 12708617

[B31] Tritsch N. X. Rodríguez-Contreras A. Crins T. T. Wang H. C. Borst J. G. Bergles D. E. (2010). Calcium action potentials in hair cells pattern auditory neuron activity before hearing onset. Nat. Neurosci. 13, 1050–1052. 10.1038/nn.2604 20676105 PMC2928883

[B32] Tritsch N. X. Yi E. Gale J. E. Glowatzki E. Bergles D. E. (2007). The origin of spontaneous activity in the developing auditory system. Nature 450, 50–55. 10.1038/nature06233 17972875

[B33] Zander J. F. Münster-Wandowski A. Brunk I. Pahner I. Gómez-Lira G. Heinemann U.. (2010). Synaptic and vesicular coexistence of VGLUT and VGAT in selected excitatory and inhibitory synapses. J. Neurosci. 30, 7634–7645. 10.1523/jneurosci.0141-10.2010 20519538 PMC6632366

